# Malignant mixed germ cell tumour of ovary- an unusual combination and review of literature

**DOI:** 10.1186/s13048-014-0091-5

**Published:** 2014-11-04

**Authors:** Lajya Devi Goyal, Sharanjit Kaur, Kanwardeep Kawatra

**Affiliations:** Department of Obstetrics and Gynaecology, Guru Gobind Singh Medical College, Faridkot, 151203 Punjab India; Department of Obstetrics and Gynaecology Nursing, University College of Nursing, Baba Farid University of Health Sciences, Faridkot, 151203 Punjab India; Department of Pathology, Christian Medical College, Ludhiana, 141001 Punjab India

**Keywords:** Malignant mixed germ cell tumour, Endodermal sinus tumour, Teratoma, Embryonal cell carcinoma

## Abstract

**Abstract:**

Mixed germ cell tumours of the ovary are malignant neoplasms of the ovary comprising of two or more types of germ cell components. Most of the malignant mixed germ cell tumours consists of dysgerminoma accompanied by endodermal sinus tumours, immature teratoma or choriocarcinoma. There are only few case reports of mixed germ cell tumours with different combinations of malignant components.

We report a very rare case of mixed germ cell tumours consisted of malignant components of endodermal sinus tumour, emryonal carcinoma, and benign component of teratomatuos and trophoblastic differentiation. This is the first case report in the literature with both benign and malignant component of type described to best of our knowledge.

Patient was an 18 year old girl, who presented with pain abdomen, abdominal mass and irregular bleeding. Ultrasound and CT scan showed a huge mass with solid and cystic component. Tumour markers i.e alpha feto- protein (AFP), human chorionic gonadotropin (hCG), lactate dehydrogenate (LDH) and Ca-125 were raised. We performed fertility sparing surgery by preserving one ovary, tube and uterus.

**Conclusion:**

Malingnant mixed germ cell tumours of ovary are highly aggressive neoplasm and early intervention and fertility sparing surgery is required for any adolescent girl presenting with rapidly enlarging pelvic mass.

## Background

Ovarian germ cell tumours arise from primordial germ cell derived from the embryonal gonads. Malignant germ cell tumour comprise less than 5% of all ovarian neoplasms. The incidence range from 1 to 6% in west and from 8 to 19% in Asia [1]. The most common form of malignant germ cell tumours are dysgerminoma (80%), endodermal sinus tumour (EST) (70%), and immature teratoma (53%) reported in a series [2]. Embryonal carcinoma, choriocarcinoma and polyembryoma are very rare type of germ cell tumour. Malignant mixed germ cell tumour is a type of tumour that consists of two or more malignant germ cell component. Most common combination reported is dysgerminoma and EST [2] and rarer component include embryonal carcinoma and immature terotoma [3,4]. Tumour markers such as AFP, hCG and LDH contribute to the diagnosis, prognosis and follow-up of the disease. We report a case of very rare mixed germ cell tumour consisted of both malignant and benign component i:e EST, embryonal carcinoma, mature teratomatuos components and trophoblastic differentiation. There are only few case reports of mixed germ cell tumour with different combinations of malignant components but this is the first case report in the literature with both benign and malignant component of type described to the best of our knowledge.

## Case report

An 18 year old girl presented with chief complaint of abdominal mass and pain of one month duration. She also complained of fever and poor appetite. Her menstrual history revealed that she had experienced menarche at the age of 12 and her cycles were regular with normal flow in the past but had irregular bleeding in last two cycles. Her physical examination revealed severe pallor and pedal edema. Her vital signs showed tachycardia (pulse rate 120/min), blood pressure 100/70 mm Hg and respiratory rate 18/min. On abdominal examination a huge mass up to the level of xiphisternum could be palpated. There was no guarding or rebound tenderness.

Investigations revealed haemoglobin 4.9 gm/dl, total count 7700, platelet count 437 × 10^3^ and on peripheral blood film there was microcytic hypochromic type of anemia. Serum biochemistry was normal. USG revealed a huge solid cystic mass occupying the whole abdomen. Right ovary was not separately visualised from the mass but left ovary was normal looking. There was no evidence of free fluid in abdomen. CT scan revealed no retroperitoneal lymphadenopathy. Tumour markers levels were CA-125 -259.3 IU/ml, Carcinoembroyonic antigen (CEA) 4.3 ng/ml alpha feto protein (AFP) 489.9 ng/ml, human chorionic gonadotropic levels (hCG) 3751.5 IU/ml and Lactate dehydrogenate (LDH) 3600 IU/ml.

Intraoperatively there was a huge mass arising from right sided ovary with intact capsule. There was no free fluid in the abdominal cavity and peritoneal washings were taken. Abdominal cavity was explored and there was no evidence of malignant disease elsewhere. Leftsided ovary and uterus was normal looking. Tumour was removed and biopsy was taken from left ovary and infracolic omentectomy and pelvic and paraaortic lymhphadenectomy was done for staging of the tumour. Frozen section could not be done as the machine was out of order.

**Figure 1:**
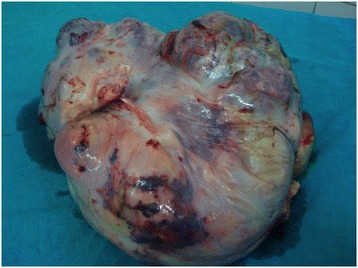
**Photograph showing gross tumour.**

**Figure 2:**
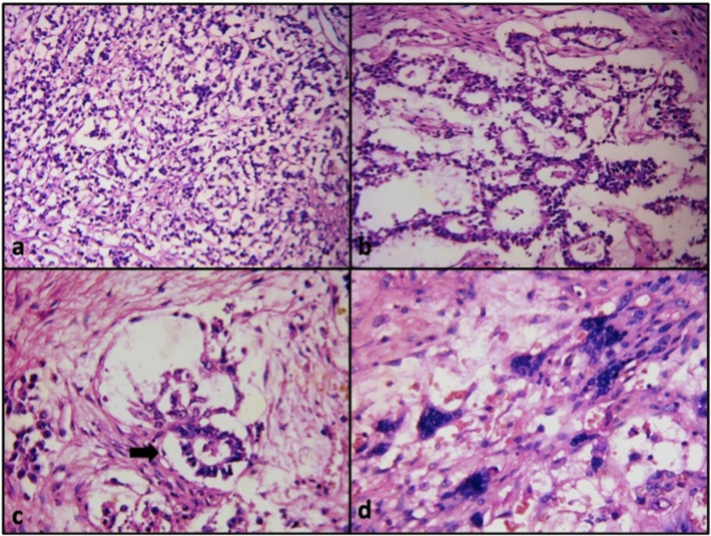
**Yolk sac tumour.** Yolk sac tumour showing reticular **(a)** and microcystic **(b)** areas with Schiller-Duval bodies **(c)**. showingmultinucleated trophoblastic giant cells **(d)**.

**Figure 3:**
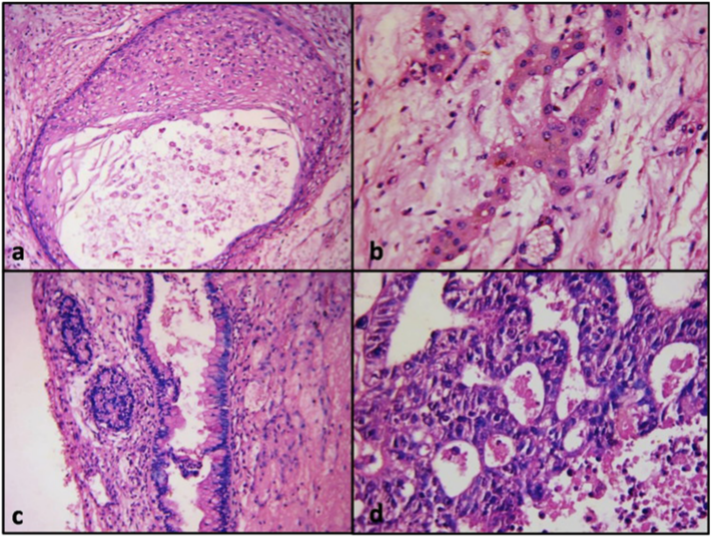
**Mixed germ cell tumour**. Showing mature teratomatous components in the form of squamous islands **(a)**, cystic spaces lined by mucinous epithelium **(c)** and hepatocytes **(b)**. Some areas also showed embryonal carcinoma **(d)**.

On gross examination (Figure 1) tumour measured 25 × 24 × 11 cm and weighed 4800 gms. External surface was smooth and bosselated with an intact capsule. Serial cut sections revealed a tumour with solid and cystic variegated cut surface showing dark-brown, grey-brown, myxoid and necrotic areas. Microscopy showed a germ cell tumour of variable composition. Predominant component was that of yolk sac tumour showing reticular (Figure 2a) and microcystic (Figure 2b) areas with Schiller-Duval bodies (Figure 2c). Several multinucleated trophoblastic giant cells were also present (Figure 2d). Along with this, there were mature teratomatous components in the form of squamous islands (Figure 3a), cystic spaces lined by mucinous epithelium (Figure 3c) and hepatocytes (Figure 3b). Some areas also showed embryonal carcinoma (Figure 3d). No extra capsular invasion was seen. Lymph nodes and omentum were also free of tumour.

Final histopathological diagnosis was: Mixed germ cell tumour with components of yolk sac tumour, mature teratoma, embryonal carcinoma and trophoblastic giant cells.

Patient was discharged in satisfactory condition and received four cycles of bleomycin, etoposide and cisplatin combination chemotherapy. Patient is on follow up and on 3 and 6 month follow up patient did not show any evidence of recurrence.

## Discussion

Rare combination of uncommon germ cell components, huge size and capsule being intact, very high levels of tumour markers makes this case unusual.

Most mixed germ cell tumour consist of combination of dysgerminoma with endodermal sinus tumour accounting for one-third of the cases [2]. Other combination include choriocarcinoma and immature teratoma in decreasing order of frequency. Cases of mixed germ cell tumour of endodermal sinus tumour and emryonal carcinoma are rare and tumours with component of benign variety of trophoblastic and mature teratomatous combination are exceptional. Recently a rare combination of mixed germ cell and granulose cell tumour has been reported in the literature [5].

The average age of presentation of germ cell tumour is 13.8 years (4–27 years) [6]. Most common clinical presentation includes abdominal mass with or without abdominal pain or fever. Embryonal carcinoma may secrete estrogen and can present with precocious puberty or irregular vaginal bleeding [7]. The gross appearance varies according to the individual constituents of the tumour. Imaging modalities can be used to establish the diagnosis but different types of tumour may show overlapping features and the definitive diagnosis is made by histopathology. In this case very high levels of tumour markers AFP and hCG could be attributed to EST and embyonal carcinoma, but we could not find any evidence of dysgerminoma component on histopathology as LDH was also highly raised. Immunohistochemistry is a wide-used biological technique that help in the diagnosis and development of new management modalities [8,9]. Trinh DT et al. described the utility of CD117, CD133, SALL4, OCT4, TCL1 and glypican-3 in malignant germ cell tumors of the ovary [9].

Ovarian endodermal sinus tumours are highly aggressive but with surgery and combination chemotherapy, the five year survival with stage 1 tumours and more advanced disease are 92% and 29% to 44% respectively [10]. Embryonal carcinoma of ovary is usually present along with other components. Survival in embryonal carcinoma the first reported series of 15 patients was 39%, with 50% of stage I patients being disease free at 3.75 to 15 years post-surgery and chemotherapy [11]. More recent data illustrate the improved survival achievable with conservative surgery and combination chemotherapy, with survival figures of 98% and 94% for early and advanced stage tumours [12]. Although standard prognostic system for ovarian germ cell tumour is currently unavailable but poor prognostic indicators include large size, unfavourable histological type, and advanced stage at presentation. Elevation of both AFP and hCG levels is a strong predictor of poor survival shown in a recent analysis [13]. Unilateral oophorectomy and surgical staging is the minimal surgery in ovarian germ cell tumour if conservation of fertility is of concern. Preservation of fertility is recommended even in metastatic disease as these tumours are highly sensitive to chemotherapy. Hysterectomy and bilateral salpingioophorectomy do not change the outcome [8]. All patients except those with FIGO stage 1a require combination chemotherapy. Usually 3 to 4 course of combination chemotherapy containing bleomycin, etoposide and cisplatin (BEP) or vincristine, dactinomycin, and cylophosphamide (VAC) are commonly used. The serum markers, may become negative during chemotherapy, but this may reflect regression of only a particular component of the mixed lesion. Therefore, in these patients a second-look laparotomy may be indicated if there is residual disease following chemotherapy.

## Conclusion

Malingnant mixed germ cell tumours of ovary are highly aggressive neoplasm and early intervention and fertility sparing surgery is required for any adolescent girl presenting with rapidly enlarging pelvic mass.

### Consent

Written informed consent was obtained from the patient for the publication of this report and any accompanying images.
